# Predictors of excess fluid volume in hemodialysis patients: an observational study

**DOI:** 10.1590/0034-7167-2022-0816

**Published:** 2024-05-03

**Authors:** Maria Isabel da Conceição Dias Fernandes, Jéssica Dantas de Sá Tinôco, Renata Marinho Fernandes, Juliana Barbosa da Silva, Anna Thays Dias Almeida, Cecília Maria Farias de Queiroz Frazão, Marcos Venícius de Oliveira Lopes, Ana Luisa Brandão de Carvalho Lira

**Affiliations:** I Universidade Federal do Rio Grande do Norte. Natal, Rio Grande do Norte, Brazil; II Universidade do Estado do Rio Grande do Norte. Caicó, Rio Grande do Norte, Brazil; III Universidade Federal de Pernambuco. Recife, Pernambuco, Brazil; IV Universidade Federal do Ceará. Fortaleza, Ceará, Brazil

**Keywords:** Nursing, Edema, Renal Dialysis, Risk Factors, Observational Study, Enfermagem, Edema, Diálise Renal, Fatores de Risco, Estudo Observacional, Enfermería, Edema, Diálisis Renal, Factores de Riesgo, Estudio Observacional

## Abstract

**Objectives:**

to assess risk factors for excess fluid volume in hemodialysis patients.

**Methods:**

a retrospective case-control study was conducted. A total of 392 patients (196 cases and 196 controls) from two hemodialysis centers were included. Sociodemographic data and 23 risk factors for excess fluid volume were assessed using a data collection form. Data were analyzed using a multivariate logistic regression model.

**Results:**

the insufficient knowledge (OR=2.06), excessive fluid intake (OR=2.33), inadequate fluid removal during hemodialysis (OR=2.62) and excessive sodium intake (OR=1.91) risk factors may increase the chance of occurrence of excess fluid volume in hemodialysis patients by approximately two times. Education level (OR=0.95) and age (OR=0.97) are protective factors for excessive fluid volume.

**Conclusions:**

knowing these risk factors may help nurses with accurate and rapid diagnostic inference of the risk of excessive fluid volume.

## RESUMO

**Objetivos:**

avaliar os fatores de risco para o excesso de volume de líquidos em pacientes em hemodiálise.

**Métodos:**

estudo retrospectivo caso-controle. Foram incluídos 392 pacientes (196 casos e 196 controles) de dois centros de hemodiálise. Dados sociodemográficos e 23 fatores de risco para excesso de volume de líquidos foram avaliados por meio de formulário de coleta de dados. Os dados foram analisados por meio de modelo de regressão logística multivariada.

**Resultados:**

os fatores de risco conhecimento insuficiente (OR=2,06), ingestão excessiva de líquidos (OR=2,33), retirada inadequada de líquidos durante hemodiálise (OR=2,62) e ingestão excessiva de sódio (OR=1,91) podem aumentar a chance de ocorrência de excesso de líquidos volume em pacientes em hemodiálise em aproximadamente duas vezes. Escolaridade (OR=0,95) e idade (OR=0,97) são fatores de proteção para volume excessivo de líquidos.

**Conclusões:**

conhecer esses fatores de risco pode auxiliar enfermeiros na inferência diagnóstica precisa e rápida do risco de volume excessivo de líquidos.

## RESUMEN

**Objetivos:**

evaluar los factores de riesgo del exceso de volumen de líquido en pacientes en hemodiálisis.

**Métodos:**

estudio retrospectivo de casos y controles. Se incluyeron 392 pacientes (196 casos y 196 controles) de dos centros de hemodiálisis. Se evaluaron datos sociodemográficos y 23 factores de riesgo de exceso de volumen de líquido mediante un formulario de recolección de datos. Los datos se analizaron mediante un modelo de regresión logística multivariado.

**Resultados:**

factores de riesgo conocimiento insuficiente (OR=2,06), ingesta excesiva de líquidos (OR=2,33), retirada inadecuada de líquidos durante la hemodiálisis (OR=2,62) e ingesta excesiva de sodio (OR=1,91) pueden aumentar aproximadamente dos veces la posibilidad de que se produzca un exceso de volumen de líquido en pacientes en hemodiálisis. La educación (OR=0,95) y la edad (OR=0,97) son factores protectores del volumen excesivo de líquido.

**Conclusiones:**

conocer estos factores de riesgo puede ayudar a las enfermeras a realizar una inferencia diagnóstica precisa y rápida del riesgo de volumen excesivo de líquidos.

## INTRODUCTION

Fluid overload stands out among the main changes resulting from end-stage renal disease (ESRD)^(1-2)^. Physiologically, patients with declining renal function lose the ability to eliminate excess fluids and sodium and, consequently, have an increased tendency to gain weight between hemodialysis sessions^(3)^.

In Brazil, the prevalence of fluid overload in hemodialysis patients is greater than 80%^(4)^, standing out as the most cited problem^(5)^. In a study with a large retrospective sample, with 5,081 adult hemodialysis patients in 23 dialysis centers in Brazil, it was identified that fluid overload was observed in 45% of patients^(6)^.

The international stage is similar to the Brazilian reality, with a considerable proportion of hemodialysis patients suffering from fluid overload. Internationally, a retrospective cohort with 38,614 hemodialysis patients observed that fluid overload is prevalent and considered a major predictor of mortality^(7-8)^.

Fluid overload is associated with cardiovascular, such as hypertension, heart failure and left ventricular hypertrophy, in addition to respiratory problems and sleep disorders, directly affecting patients’ quality of life and survival^(1,9-12)^. Thus, it is associated with increased hospital admission rates, morbidity, and mortality^(7,13-14)^.

Therefore, volume control and assessment can be important to avoid or reduce complications, and for improving hemodialysis patients’ survival^(12)^. Objective measurements of fluid status are needed to support healthcare staff in identifying and treating fast and accurately fluid overload^(8)^. Thus, knowing the set of risk factors that predict excess fluid volume is essential for maintaining these patients’ health. Despite that, in the scientific literature^(15-17)^, these factors are studied in isolation, and some reports have identified fragile methodologies.

Furthermore, standardized language system that brings together phenomena of interest to nursing, NANDA International, does not include in its current version a nursing diagnosis that includes risk factors for fluid overload^(18)^. Therefore, the question arises: which risk factors increase the chances of fluid overload occurring in hemodialysis patients?

In view of these gaps in knowledge, there is a need to clinically validate the main risk factors for excessive fluid volume in hemodialysis patients to assist nurses in accurately predicting them for this clientele. Thus, it is believed that this study will advance nursing knowledge by confirming the set of risk factors that increase vulnerability to excess fluids in hemodialysis patients. This knowledge contributes to developing qualified care for these individuals, minimizing health costs, in addition contributing to nursing science and standardized language systems with phenomena specific to nursing.

## OBJECTIVES

To assess risk factors for excess fluid volume in hemodialysis patients.

## METHODS

### Ethical aspects

The study was conducted in accordance with national and international ethics guidelines and approved by the Research Ethics Committee of the *Universidade Federal do Rio Grande of Norte*, whose opinion is attached to this submission. The Informed Consent Form was obtained from all individuals involved in the study in writing.

### Study design, period and place

A retrospective case-control study was performed at two hemodialysis reference centers located in northeastern Brazil in 2018. This study meets the quality standards established in the STrengthening the Reporting of OBservational studies in Epidemiology (STROBE) instrument for health research production.

### Sample, inclusion and exclusion criteria

The population of this study was composed of 600 patients who underwent hemodialysis therapy at two hemodialysis centers. The sample was calculated according to the following specifications: a 95% confidence level; an 80% power; an equal ratio between the number of cases and controls (r = 1); a proportion of individuals exposed in the control group of 50% (p2 = 0.50); an Odds Ratio (OR) of 1.8 for developing excess liquid volume (OR = 1.8); a mean proportion of the occurrence of the main risk factor for the study of = 0.57; and a proportion of individuals exposed in the case group of p1 = 0.64.

We adopted p2 = 0.5 because it is a strategy that maximizes sample size and demonstrates initial ignorance of the true proportion of risk factors, in addition to considering the possibility of multiple risk factors that could present different proportions. The proportion of exposed individuals is given by their relationship with the adopted OR of the initially p2 proportion defined. The mean proportion is the mean between p1 and p2 according to the adopted formula. Thus, the sample comprised 196 patients in each group (196 cases and 196 controls), totaling 392 participants.

Patients were classified as cases (patients with excess fluid volume) when they had an interdialytic weight gain greater than 3.5% of dry weight and edema. Controls (patients without excess fluid volume) were classified into this group when they had an interdialytic weight gain of less than 3.5% of dry weight and no edema.

As a theoretical basis for the division between cases and controls, a diagnostic accuracy survey was used, which identified accurate signs and symptoms to differentiate hemodialysis patients with and without excessive fluid volume^(4)^. The survey found that edema has a sensitivity of 92.7% for diagnosing fluid overload in these patients. Additionally, interdialytic weight gain assessment was also considered, since, before the visible appearance of edema, there may be an accumulation of three to five liters of fluid, which is objectively expressed from sudden weight gain. This assessment assists in confirming that patients are really free of excess fluid^(19)^. In this regard, to minimize selection bias, which would impair the screening of cases and controls in this study, interdialytic weight gain assessment combined with edema identification was adopted.

The estimate of maximum weight between dialyses can be calculated from dry weight assessment. Dry weight is defined as a theoretical normovolemic state used as a target weight for patients after dialysis treatment, being considered the lowest weight tolerated by patients without developing hypotension and cramps. When weight gain acquired during the interdialytic period exceeds 3.5% of the ideal dry weight, patients have excess fluid^(19)^.

Individuals with ESRD, older than 18 years, under hemodialysis for a period of at least three months for three times a week for four hours were included. In both groups, individuals who could not respond to items of the data collection instrument were excluded.

After applying the eligibility criteria, each patient was assessed for pre-dialysis weight, estimated dry weight and edema, and divided into cases and controls. All patients included within the criteria were weighed before starting dialysis to check pre-dialysis weight, on a single digital scale (Toledo^®^), only by the study researcher. Then they were assessed for the existence of edema by assessing Godet’s sign in the lower limbs and assessing the presence of periorbital edema. Additionally, the estimated dry weight was checked in the medical records. Then, a simple rule of three calculation was performed to estimate whether interdialytic weight gain exceeded dry weight.

Consecutive sampling was used to recruit participants. Patients were divided into cases (196 patients) and controls (196 patients) in a 1:1 manner, with subsequent data collection.

### Study protocol

Data collection was carried in 2018 using a data collection instrument with sociodemographic data (age, gender, income, ethnicity, religion, and education level) and 23 risk factors for excess fluid volume: increased dialysate sodium concentration; absence in the hemodialysis session; insufficient water assessment; low self-efficacy for fluid restriction; comorbidities; insufficient knowledge; renal function decline; decreased urinary volume; inflammatory state; hospital admission; age ≥ 60 years; altered Body Mass Index; excessive fluid intake; excessive protein intake; excessive sodium intake; low Kt/V index; low serum albumin level; elevated serum phosphorus level; decreased serum lymphocyte level; inadequate fluid removal during hemodialysis; thirst; use of antihypertensive drugs; and xerostomia.

For the construction of this data collection instrument, the conceptual and operational definitions of each of the 23 risk factors were previously developed^(20)^. Regarding the assessment of insufficient knowledge, the researcher asked the interviewee questions about the definition, signs/symptoms, consequences and possible causes of excessive fluid volume in hemodialysis patients. If the respondent did not know how to answer the questions or answered incorrectly, the factor was marked as present. Regarding excessive fluid intake, the examiner asked the respondent about the amount of fluid ingested in the 24 hours and the amount of urine eliminated on the same day. If fluid intake was higher than the maximum recommended (500 ml added to the residual diuresis value), the factor would be present.

The instrument was pretested on a group corresponding to 10% of the sample. No problems were experienced during pretest, and no changes were made. Thus, pretest participants were included in the final sample.

Data were obtained from primary sources through direct interactions with patients. The presence or absence of each risk factor for excess fluid volume was identified throughout the interviews.

For data collection, eight collaborators participated, and all were previously trained on the data collection procedures. In the data collection process, only the main researcher knew about the division of patients into cases and controls, and collaborators and patients were blinded.

### Analysis of results, and statistics

Data were organized in a Microsoft Office Excel spreadsheet and processed using the R version 3.1.1 statistical package. Descriptive statistics were used in categorical socioeconomic data analysis. The Fisher-Freeman-Halton test was used to assess the similarity between case and control groups. This test is used as a generalization of Fisher’s test, allowing an analysis of the difference in proportions between variables with more than two categories. Measures of central tendency and dispersion of numerical variables were obtained, and the Mann-Whitney test was also applied to assess the similarity of mean ranks between groups in relation to quantitative variables that do not follow a normal distribution. Mean ranks represent orderings of variable values similar to a measure of central tendency.

Association analysis between dichotomous variables was carried out using Fisher’s Exact test, and the magnitude of effect of risk factors on nursing diagnosis was based on OR. In this case, values above 1 for OR represent an increased chance of the diagnosis occurring in the presence of the risk factor. Additionally, backward stepwise regression, a multivariate logistic regression model, was performed to confirm the causal relationships between the sets of risk factors and the occurrence of excess fluid volume. This model is based on the entry of all variables with significance less than or equal to 0.2 in the initial model, followed by the sequential removal of each variable from the model according to the highest p-value in the set, until a model consisting of variables with p-value is obtained <0.05.

In the multivariate logistic regression model, the Omnibus test was applied to verify the model’s significance. The statistical significance of this test demonstrates that the model is capable of correctly classifying the subjects in the sample. The adequacy of each variable included in the regression model was defined by a statistical significance for Wald’s chi-square test. The Hosmer-Lemeshow test was applied to verify the goodness of fit, indicating an adequate adjustment of model based on p-value > 0.05. Nagelkerke’s R^2^ was calculated to verify the model’s predictive capacity. This coefficient represents a percentage of explained variation in the probability of identifying a nursing diagnosis based on the presence of the identified risk factor. The beta regression coefficient (coef.) and standard error (S.E.) of the beta coefficient were used in the OR calculation. For inclusion in the logistic regression model, all risk factors and sociodemographic variables with statistical significance ≤ 0.2 in the bivariate analysis were chosen. Variables included in the multivariate model were analyzed for multicollinearity using the variance inflation factor (VIF), which was < 5 for all variables included in the final adjustment. Therefore, there were no variables with multicollinearity. The absence of multicollinearity is an essential assumption for the application of multivariate regressions.

## RESULTS

All 392 potentially eligible individuals were confirmed to be eligible, included in the study and assessed. According to Table 1, the individuals in the case group were predominantly female, and those in the control group were predominantly male. In both groups, most were brown and reported having a religion. The case group had a median of 8.5 years of education level, while the control group had a median of 10 years. The case and control groups were homogeneous concerning the variables mentioned above (p > 0.05).

**Table 1:** Characteristics of the case and control subjects (N = 392)

Variables			Groups					Total			OR 95%CI		X^1^	*p* value^2^
			Case (n = 196)		Control (n = 196)									
			n	%	n		%	n		%				
Gender														
Female			101	51.5	87		44.4	188		48.0	1.33		2.00	0.157
Male			95	48.5	109		55.6	204		52.0	0.89-1.98			
Ethnicity														
Brown			104	53.1	103		52.6	207		52.8				
White			53	27.0	67		34.2	120		30.6	--		4.24	0.120
Black			39	19.9	26		13.3	65		16.6				
Religion														
Yes			118	60.2	118		60.2	236		60.2	1,00		0.00	1.000
No			78	39.8	78		39.8	156		39.8	0.67-1.50			
**Variables**	**Groups**	**Minimum/Maximum**		**Median**	**IQR**	**K-S test^1^**					**Mann-Whitney test**			
						**D**		**Df**	***p* value**		**MR**	**U**		***p* value**
Age (years)	Case	20/86		51.0	22	0.073		196	0.014		177.5	15488.5		0.001
	Control	18/89		56.0	21	0.051		196	0.200		215.5			
Education level	Case	0/20		8.5	08	0.143		196	<0.001		186.8	17312.5		0.088
	Control	0/23		10.0	08	0.149		196	<0.001		206.2			
Family income	Case	0/20		2.0	02	0.278		196	<0.001		181.7	16305.0		0.007
	Control	0/60		2.0	03	0.310		196	<0.001		211.3			

1 Kolmogorov-Smirnov test;

2 Fisher-Freeman-Halton test; MR = mean ranks.

A significant difference was identified between groups regarding age (mean ranks: 177.5 vs. 215.5; Mann-Whitney U = 15488.5; p = 0.001). The case group comprised individuals who were younger than those in the control group. The case and control groups had statistical differences of family income (mean ranks: 181.7 vs. 211.3; Mann-Whitney U = 15488.5; p = 0.007), as individuals in the case group had lower incomes than those in the control group.

According to Table 2, four risk factors were statistically associated with excess fluid volume in hemodialysis patients: age ≥ 60 years (p = 0.002); inadequate fluid removal during hemodialysis (p < 0.001); excessive fluid intake (p <0.001); and excessive sodium intake (p = 0.029). The inadequate fluid removal during hemodialysis (OR = 2.44; 95%CI: 1.61 - 3.67), excessive fluid intake (OR = 2.39; 95%CI: 1.59 - 3.59) and excessive sodium intake (OR = 1.91; 95%CI: 1.06 - 3.45) risk factors were related to an almost two-fold increased risk of developing excessive fluid volume. An age of ≥ 60 years was a protective factor for developing excess fluid volume (OR = 0.52; 95%CI: 0.34 - 0.79), indicating that increased risk of excessive fluid volume is inversely related to aging.

**Table 2:** Risk factors for excess fluid volume in hemodialysis patients (N = 392)

Risk factors	Case		Control		Total		Odds Ratio 95%CI	X^2^	*p* value^1^
	n	%	n	%					
Increased dialysate sodium concentration	00	0.0	02	1.0	02	1.0	--	--	0.499
Absence in the hemodialysis session	18	9.2	22	11.2	40	10.2	0.80 0.41-1.54	0.44	0.505
Insufficient water assessment	133	67.9	144	73.5	277	70.7	0.76 0.49-1.18	1.49	0.222
Low self-efficacy for fluid restriction	08	4.1	07	3.6	15	3.8	1.15 0.41-3.23	0.07	0.792
Comorbidities	118	60.2	126	64.3	244	62.2	0.84 0.56-1.26	0.69	0.405
Insufficient knowledge	52	26.5	38	19.4	90	23.0	1.50 0.93-2.41	2.83	0.093
Decline in renal function	196	100	196	100	392	100	--	--	--
Decreased urine volume	147	75.0	137	69.9	284	72.4	1.29 0.83-2.01	1.28	0.258
Inflammatory state	11	5.6	11	5.6	22	5.6	0.99 0.38-2.60	0.00	1.000
Hospital admission	12	6.1	18	9.2	30	7.7	0.64 0.30-1.38	1.30	0.254
Age ≥ 60 years	54	27.6	83	42.3	137	34.9	0.52 0.34-0.79	9.44	0.002
Altered Body Mass Index	102	52.0	111	56.6	213	54.3	0.83 0.56-1.24	0.83	0.361
Excessive fluid intake	114	58.2	72	36.7	186	47.4	2.39 1.59-3.59	18.04	<0.001
Excessive protein intake	167	85.2	157	80.1	324	82.7	1.43 0.84-2.42	1.78	0.182
Excessive sodium intake	176	89.8	161	82.1	337	86.0	1.91 1.06-3.45	4.76	0.029
Low Kt/V index	28	14.3	32	16.3	60	15.3	0.85 0.49-1.48	0.31	0.575
Low serum albumin level	--	--	--	--	--	--	--	--	--
High serum phosphorus level	88	44.9	77	39.3	165	42.1	1.26 0.84-1.88	1.27	0.260
Decreased serum lymphocyte level	57	29.1	64	32.7	121	30.9	0.85 0.55-1.30	0.59	0.444
Inadequate fluid removal during hemodialysis	133	67.9	91	46.4	224	57.1	2.44 1.61-3.67	18.37	<0.001
Thirst	187	95.4	189	96.4	376	95.9	0.77 0.28-2.11	0.26	0.610
Use of antihypertensive drugs	31	15.8	19	9.7	50	12.8	1.75 0.95-3.22	3.30	0.069
Xerostomia	184	93.9	180	91.8	364	92.9	1.36 0.63-2.96	0.61	0.433

CI - confidence interval;

1 Fisher’s Exact test.

Table 3 shows the results of the backward stepwise regression used to identify and confirm the causal relationships between sets of risk factors and the study outcome. Risk factors and sociodemographic variables with p ≤ 0.2 were included in the bivariate analysis.

**Table 3:** Logistic regression model for the risk of excess fluid volume in hemodialysis patients (N = 392)

Variables	Coef.	S.E.	X^2^	df	Sig.	OR	95%CI	
Insufficient knowledge	0.72	0.29	6.33	1	0.012	2.06	1.17	3.61
Excessive fluid intake	0.85	0.22	14.29	1	<0.001	2.33	1.50	3.61
Inadequate fluid removal during hemodialysis	0.96	0.22	18.71	1	<0.001	2.62	1.69	4.06
Education level	-0.05	0.02	4.68	1	0.031	0.95	0.91	0.99
Age	-0.03	0.01	13.81	1	<0.001	0.97	0.96	0.98
Constant	0.84	0.51	2.70	1	0.100	2.33		
Adjustment measures				df	Sig.			
Hosmer-Lemeshow test			10.28	8	0.246			
Omnibus test			58.53	5	<0.001			
Nagelkerke’s R^2^			0.185					

*Coef. - beta regression coefficient; Sig. - significance; OR - Odds Ratio; CI - confidence interval.*

The insufficient knowledge (OR = 2.06; 95%CI: 1.17 - 3.61; X^2^ = 6.33; p = 0.012), excessive fluid intake (OR = 2.33; 95%CI: 1.50 - 3.61; X^2^ = 14.29; p < 0.001) and inadequate fluid removal during hemodialysis (OR = 2.62; 95%CI: 1.69 - 4.06; X^2^ = 18.71; p < 0.001) risk factors may increase the risk of developing excess fluid volume in hemodialysis patients. These factors increase the chance of nursing diagnosis occurring by approximately two times. Education level (OR = 0.95; 95%CI: 0.91 - 0.99; X^2^ = 4.68; p = 0.031) and age (OR = 0.97; 95%CI: 0.96 - 0.98; X^2^ = 13.81; p < 0.001) are protective factors that may decrease the risk of developing excess fluid volume, with a risk reduction of around 5% for each year of age or education. These claims were corroborated by the Omnibus test (p < 0.001) and chi-square test (p < 0.05). Furthermore, the observed and expected frequencies in the final model showed no significant differences according to the Hosmer-Lemeshow test (p = 0.246), indicating that the goodness of fit was achieved.

## DISCUSSION

In this study, insufficient knowledge was an important risk factor for excess fluid volume among hemodialysis patients. Studies confirm the findings of this study, when they report that having insufficient knowledge concerning dietary and water intake can cause excessive hydration and high weight gain between hemodialysis sessions^(21-22)^. In contrast, when these patients have a higher level of knowledge, greater self-management of prescribed restrictions and less liquid consumption occur, with lower chances of water overload^(22)^.

In this study, education, when high, can be considered a protective factor for excess fluid volume in hemodialysis patients. The median education was lower in the case group, with water overload. Thus, it is inferred that the lower the educational level, the greater the chance of developing excess fluid volume. And the higher the education level, the lower the chance of developing excess fluid volume.

About this aspect, the literature shows a higher intake of sodium and fluids, and a low adherence to restriction among patients with less education than those with higher education^(23-24)^. From this perspective, a low education level tends to negatively influence knowledge this patients, resulting in the development of excess fluid volume. Therefore, increased knowledge in this group of patients can lead to better self-care, disease management, and adherence to healthy behaviors^(25)^.

Excessive sodium intake is another important factor for the risk of excess fluid volume in this study. A study on excess fluid volume in hemodialysis patients confirms this finding. It was found that excessive sodium intake is a predictive factor for occurrence of water overload in this clientele, demonstrating a probability of occurrence of 87.5%^(26)^. After a meal rich in sodium, thirst increases. Consequently, individuals will increase their water intake^(27)^. Hemodialysis patients often have difficulties following a low-sodium diet set out in clinical guidelines^(17)^.

Several causes are described for the problem mentioned above, such as lack of practical knowledge about diet restrictions, low motivation, lack of social support, little feedback on sodium intake, low availability of low-sodium foods, a perception that a low-sodium diet is not tasty, and lack of feedback from health professionals^(28)^.

In this aspect, there is a need for educational measures aimed at self-care, about the disease, treatment and repercussions on health due to low adherence^(26)^. A study states that cell phone text messages are a well-accepted strategy as a counseling support for people on hemodialysis to improve eating behaviors. Participants felt supported by the messages and were motivated to adopt new eating and lifestyle habits^(29)^.

In this study, water intake restriction should also be emphasized for preventing excess fluid volume among hemodialysis patients. Nonadherence to the prescribed fluid regimen is a common problem in hemodialysis patients, and it is associated with increased morbidity and mortality and fluid overload^(15,23,30)^. Thus, educational interventions to improve patients’ self-efficacy can improve adherence to water restriction^(22)^. Additionally, a timetable distribution of fluids and individualized nutritional counseling improve adherence to the fluid restriction regimen^(31)^.

Inadequate fluid removal during hemodialysis stands out as an important factor can increase the risk of developing excess fluid volume. Fluid assessment performed with insufficient frequency (> two weeks) stands out among the precipitants^(27)^. When health professionals do not constantly assess patients’ fluid status, the amount of fluid removed during hemodialysis may not be adequate. Moreover, some actions taken by patients, such as excessive interdialytic weight gain, also influences inadequate fluid removal. When a patient gains excessive weight between dialysis sections, the body cannot support removing a large volume of fluids in a short time (3-4 hours).

Fluid management and assessment in hemodialysis patients is a major challenge for professionals working in nephrology. Over time, a series of alternatives were used to fluid overload status in this clientele, such as fluid status clinical assessment, hemodynamic stability verification, bioimpedance use, or cardiac and vascular biomarker assessment. Currently, it is considered ideal to assess sodium and fluid balance in these patients from dialysate ultrafiltration and sodium adjustment, in addition to guiding sodium restrictions and weight gain between dialyses^(1)^. Additionally, a study highlighted that switching from conventional hemodialysis to daily hemodialysis was associated with better fluid overload control and a lower risk of death^(32)^.

Regarding age, we also found that individuals aged 60 or older had a decreased risk of excess fluid volume. Thus, increased risk of excessive fluid volume in these patients is inversely related to aging. Evidence indicates that younger participants were more likely to report diet management problems and low self-efficacy to restrict sodium intake, confirming the above claim. Thus, these individuals had a higher mean interdialytic weight gain^(23,33)^.

Nurses should mainly observe the presence of modifiable risk factors and implement interventions with the support of a multidisciplinary team, such as estimating patients’ dry weight to accurately and gradually remove fluids during hemodialysis, the time spent on hemodialysis to achieve optimal ultrafiltration and sodium removal, as well as planning educational activities and training to contribute to greater adherence to diet, significantly reducing sodium intake and fluid restriction. Proper implementation of these strategies would help reduce volume overload in hemodialysis patients^(34-35)^. Additionally, more objective measurements of patients’ fluid status are needed to further support healthcare professionals in identifying and treating fluid overload^(8)^.

### Study limitations

The unpaired case and control groups may have affected the identification of risk factors for excess fluid volume. The matching technique in sample selection for case-control studies aims to standardize intervening variables that may interfere with the outcome studied. This problem was resolved in the analysis of sociodemographic data from the case and control groups, because they introduced themselves mostly homogeneous. Furthermore, the OR was greater than two in this study, confirming the relevance of the risk factors identified.

### Contributions to the area of nursing and public health

This study provides research data based on a high level of evidence for teaching nursing students and nurses in clinical practice. The results found in this study add knowledge and can help nurses with accurate and rapid diagnostic inference of the risk of excessive fluid volume, with the aim of reducing the chances of hemodialysis patients developing complications arising from fluid overload, which reduces the chances of hospital admission, medical expenses, and increased survival.

## CONCLUSIONS

This article summarizes and clinically validates a set of risk factors for developing excessive fluid volume in hemodialysis patients. The insufficient knowledge, excessive fluid intake, inadequate fluid removal during hemodialysis and excessive sodium intake risk factors may increase the chance of occurrence of excess fluid volume in hemodialysis patients by approximately two times. Educational level and age are protective factors may decrease the risk of developing excess fluid volume. People undergoing hemodialysis over 60 years old and with a higher education level may experience a 5% reduction in the risk of excess fluid volume.

## References

[1] Canaud B, Chazot C, Koomans J, Collins A. (2019). Fluid and hemodynamic management in hemodialysis patients: challenges and opportunities. J Bras Nefrol.

[2] Canaud B, Morena-Carrere M, Leray-Moragues H, Cristol JP. (2022). Fluid overload and tissue sodium accumulation as main drivers of protein energy malnutrition in dialysis patients. Nutrients.

[3] Cabrera C, Brunelli SM, Rosenbaum D, Anum E, Ramakrishnan K, Jensen DE (2015). A retrospective longitudinal study estimating the association between interdialytic weight gain and cardiovascular events and death in hemodialysis patients. BMC Nephrol.

[4] Fernandes MICD, Bispo MM, Leite EMD, Lopes MVO, Silva VM, Lira ALBC. (2015). Diagnostic accuracy of the defining characteristics of the excessive fluid volume diagnosis in hemodialysis patient. Rev Latino-Am Enfermagem.

[5] Brezolin CA, Lima MVR, Seidel EN, Mendonça HSL. (2018). Nursing diagnoses for hemodialytic patients: integrative review. Rev Enferm UFPI.

[6] Barra ABL, Roque da Silva AP, Canziani MEF, Lugon JR, Strogoff de Matos JP (2022). Characteristics and predictors of mortality on haemodialysis in Brazil: a cohort of 5.081 incident patients. BMC Nephrol.

[7] Hecking M, Moissl U, Genser B, Rayner H, Dasgupta I, Stuard S (2018). Greater fluid overload and lower interdialytic weight gain are independently associated with mortality in a large international hemodialysis population. Nephrol Dial Transplant.

[8] Moissl U, Fuentes LR, Hakim MI, Hassler M, Kothari DA, Rosales L. (2022). Prevalence of fluid overload in an urban US hemodialysis population: a cross-sectional study. Hemodial Int.

[9] Hao G, Lu W, Huang J, Ding W, Wang P, Wang L (2018). Predialysis fluid overload linked with quality os sleep in patients undergoing hemodialysis. Sleep med.

[10] Kaartinen K, Rautavaara J, Aitkoski A, Ahvonem J, Vilpakka M, Koistinen J (2020). Fluid overload is in independent predictor of atrial fibrillation in end-stage renal disease: a prospective study using insertable cardiac and body composition monitors. Clin Nephrol.

[11] Zhang L, Zhang X, Xue S. (2022). Left heart failure caused by capacity overload in peritoneal dialysis patients. BioMed Res Int.

[12] Sousa GR, Serra KS, Silva APS, Franco CMMA, Bonfim IM, Stuart RMB. (2021). Evidence about the occurrence of complications related to unbalanced volume of the chronic kidney patient. Saúde Coletiva.

[13] Siriopol D, Siriopol M, Stuard S, Voroneanu L, Wabel P, Moissl U (2019). An analysis of the impact of fluid overload and fluid depletion for all-cause and cardiovascular mortality. Nephrol Dial Transplant.

[14] Zoccali C, Moissl U, Chazot C, Mallamaci F, Tripepi G, Arkossy O (2017). Chronic fluid overload and mortality in ESRD. J Am Soc Nephrol.

[15] Bellomo G, Coccetta P, Pasticci F, Rossi D, Selvi A. (2015). The effect of psychological intervention on thirst and interdialytic weight gain in patients on chronic hemodialysis: a randomized controlled trial. J Ren Nutr.

[16] Lee JE, Jo IY, Lee SM, Kim WJ, Choi HY, Ha SK (2015). Comparison of hydration and nutritional status between young and elderly hemodialysis patients through bioimpedance analysis. Clin Interv Aging.

[17] Liu J, Sun F, Ma L, Shen Y, Mei X, Zhou Y. (2016). Increasing Dialysis sodium removal on arterial stiffness and left ventricular hypertrophy in hemodialysis patients. J Ren Nutr.

[18] Herdman TH, Kamitsuru S, Lopes CT. (2021). Nursing diagnosis: definitions and classifications 2021-2023.

[19] Cho MK. (2013). Effect of health contract intervention on renal dialysis patients in Korea. Nurs Health Sci.

[20] Fernandes MICD. (2018). Construção e validação do diagnóstico de enfermagem risco de volume de líquidos excessivo a partir de uma teoria de médio alcance.

[21] Lima MA, Sousa GR, Sousa AM, Felipe GF, Oliveira AS, Formiga LMF. (2014). Health education for hemodialysis patients. Rev Enferm UFPE.

[22] Parker JR. (2019). Use of an educational intervention to improve fluid restriction adherence in patients on hemodialysis. Nephrol Nurs J.

[23] Nerbass FB, Correa D, Santos RG, Kruger TS, Sczip AC, Vieira MA (2017). Perceptions of hemodialysis patients about dietary restrictions. J Bras Nefrol.

[24] Xie J, Ding S, Liu L, Liu Z, Zhang Q, Duan Y (2017). Health beliefs of salt intake among patients undergoing haemodialysis. J Ren Care.

[25] Wu SFV, Hsieh NC, Lin LJ, Tsai JM. (2016). Prediction of self-care behaviour on the basis of knowledge about chronic kidney disease using self-efficacy as a mediator. J Clin Nurs.

[26] Botelho ML, Correia MDL, Manzoli JPB, Montanari FL, Carvalho LAC, Duran ECM. (2021). Classification tree for the inference of the nursing diagnosis Fluid Volume Excess (00026). Rev Esc Enferm USP.

[27] Weiner DE, Brunelli SM, Hunt A, Schiller B, Glassock R, Maddux FW (2014). Improving clinical outcomes among hemodialysis patients: a proposal for a "volume first" approach from the chief medical officers of US dialysis providers. Am J Kidney Dis.

[28] Meuleman Y, Hoekstra T, Dekker FW, Boog PJMVD, Dijk SV. (2018). Perceived sodium reduction barriers among patients with chronic kidney disease: Which barriers are important and which patients experience barriers?. Int J Behav Med.

[29] Dawson J, Tong A, Gonzalez AM, Campbell KL, Craig JC, Lee VW. (2021). Patients’ experiences and perspectives of a mobile phone text messaging intervention to improve dietary behaviours in haemodialysis. Nutr Diet.

[30] Vijay VR, Harmeet KK. (2022). The worldwide prevalence of nonadherence to diet and fluidrestrictions among hemodialysis patients: a systematic review and meta-analysis. J Ren Nutr.

[31] Mina RJL, Lerma MB, Litan PLB, Milano AA, Mojica ADR, Malong-Consolacion CP Fluid distribution timetable on adherence to fluid restriction of patients with end-stage renal disease undergoing haemodialysis: single-blind, randomized-controlled pilot study. J Adv Nurs.

[32] Barra ABL, Roque-da-Silva AP, Vasconcellos MS, Lugon JR, Strogoff-de-Mato JP. (2020). Association between extracellular volume control and survival in patients on short daily haemodialysis. BMC Nephrol.

[33] Clark-Cutaia MN, Ren D, Hoffman LA, Burke LE, Sevick MA. (2014). Adherence to hemodialysis dietary sodium recommendations: influence of patient characteristics, self-efficacy, and perceived barriers. J Ren Nutr.

[34] Loutradis C, Sarafidis PA, Ferro CJ, Zoccali C. (2021). Volume overload in hemodialysis: diagnosis, cardiovascular consequences, and management. Nephrol Dial Transplant.

[35] Baser E, Mollaoglu M. (2019). The effect of a hemodialysis patient education program on fluid control and dietary compliance. Hemodial Int.

